# Phytochemical profile and chemosensitizing anticancer activity of *Mitragyna speciosa* and mitragynine

**DOI:** 10.1038/s41598-026-43711-5

**Published:** 2026-03-11

**Authors:** Panita Kongsila, Thidarut Boonmars, Pranee Sriraj, Jatuporn Prathumtet, Praphat Manuelo Ruengthanoo, Ratchadawan Aukkanimart

**Affiliations:** 1 Department of Thai Traditional Medicine, Faculty of Natural Resources, Rajamangala University of Technology Isan, Sakon Nakhon Campus, Sakon Nakhon, 47160 Thailand; 2 Department of Parasitology, Faculty of Medicine, Khon Kaen University, Khon Kaen, 40002 Thailand; 3 Cholangiocarcinoma Research Institute (CARI), Faculty of Medicine, Khon Kaen University, Khon Kaen, 40002 Thailand

**Keywords:** *Mitragyna speciosa*, Mitragynine, Antioxidant activity, Anticancer activity, Synergistic effect, Biochemistry, Cancer, Drug discovery, Oncology

## Abstract

This study examined the phytochemical composition, antioxidant efficacy, and cytotoxic properties of *Mitragyna speciosa* (kratom) extracts and its principal alkaloid, mitragynine. The extracts exhibited significant antioxidant properties, and chemical analysis verified mitragynine as the principal alkaloid, aligning with the distinctive phytochemical profile of *M. speciosa.* In vitro cytotoxicity studies demonstrated that both kratom extract and mitragynine suppressed cancer cell proliferation (A549, KKU213C, and HeLa cancer cell lines) in a concentration- and time-dependent manner, with mitragynine displaying greater potency compared to the crude extract, especially against cholangiocarcinoma cells. Importantly, synergistic cytotoxic effects were noted when kratom extract or mitragynine was combined with standard chemotherapeutic agents across various cancer cell lines, especially HeLa cervical cancer. These effects correlated with diminished expression of the anti-apoptotic protein BCL-2. In general, the results show that *M. speciosa* extract and mitragynine are chemosensitizers that could make standard chemotherapy work better. Additional research is necessary to clarify the molecular mechanisms involved and to confirm these effects in vivo.

**Supplementary Information:**

The online version contains supplementary material available at 10.1038/s41598-026-43711-5.

## Introduction

 Cancer remains the leading cause of mortality worldwide, surpassing ischemic heart disease and stroke^1^. The global incidence of cancer is projected to exceed 20 million new cases annually by 2025, representing a substantial public health burden. Among all cancer types, lung (1.82 million cases), breast (1.67 million cases), and colorectal cancer (1.36 million cases) are the most commonly diagnosed, while lung cancer continues to account for the highest mortality rate, followed by liver and stomach cancers. These figures underscore the urgent need for effective prevention, early detection, and improved therapeutic strategies^1^. Conventional cancer treatments such as surgery, radiation, and chemotherapy continue to face significant limitations. Chemotherapy is associated with severe side effects and the development of chemoresistance^2^. In parallel, the development of novel anticancer drugs remains costly, complex, and time-consuming. In response to these challenges, combination therapy has emerged as a promising alternative to monotherapy. By concurrently targeting multiple molecular pathways, this approach enhances therapeutic efficacy while potentially reducing drug resistance. These combinations may improve anticancer effectiveness, diminish drug resistance, and facilitate the administration of reduced doses of chemotherapeutic drugs^3^.

*Mitragyna speciosa* (Korth.) Havil., commonly known as kratom, is a medicinal plant belonging to the Rubiaceae family and is native to Southeast Asia, particularly Malaysia, Indonesia, and Thailand. In Thai traditional medicine, the leaves of kratom are used for many purposes such as treatment of abdominal pain, dysentery, diarrhea, and muscle pain. It is also used for helping people with opium withdrawal and sometimes as a sedative. Five traditional herbal formulations that contain kratom are reported in Thai traditional pharmacopoeia (Khun Sophit Bannalak). Phytochemical tests have shown that mitragynine is the main alkaloid in kratom leaves (approximately 66.2% of the total content)^4^. Researchers have reported that mitragynine has a lot of various biological effects, such as pain relief, anti-inflammatory^5^, cytotoxicity^6^, muscle relaxant effects^7^, and antidepressant-like^8^, It doesn’t seem to be very toxic to normal cells.

Moreover, both kratom extracts and mitragynine exhibit antioxidant properties and antiproliferative effects against various cancer cell lines^9^. Unlike previous studies that mainly focused on cisplatin-based combinations, this study also evaluated gemcitabine and compared crude kratom extracts with purified mitragynine. In addition, the combination effects of kratom extract and mitragynine on cholangiocarcinoma cell lines have not been previously reported, especially in comparison with lung and cervical cancer cell lines. Given the need for adjuvant strategies to improve chemotherapy efficacy and reduce drug dosage. This study aimed to characterize the chemical profile of kratom extracts and investigate their chemosensitizing effects, together with mitragynine, in combination with standard anticancer agents against KKU213C, A549, and HeLa cancer cell lines.

## Materials and methods

### Plant collection and extraction

Kratom leaves were collected from Thung Fon district, Udon Thani province, northeastern Thailand. The collection and use of *M. speciosa* Korth., Rubiaceae for this study were officially permitted by the Cannabis and Herb Institute, Rajamangala University of Technology Isan, Sakon Nakhon Campus (voucher specimen no. 2024MSgreen-UD01). The plant species was taxonomically identified and authenticated by the institute’s expert botanist Asst. Prof. Pichet Wechvitan, Ph.D., and the voucher specimen was deposited in the Herbarium Collection of the Department of Thai Traditional Medicine, Faculty of Natural Resources, Rajamangala University of Technology Isan, Sakon Nakhon Campus. The legal framework for kratom research and utilization in Thailand was established under the Narcotics Act (No. 8) B.E. 2564 (2021), which was published in the Government Gazette on May 26, 2021, and came into effect 90 days thereafter. This amendment followed the earlier Narcotics Act (No. 7) B.E. 2562 (2019), which first legalized the medical use of cannabis and kratom in Thailand.

The dried leaves were ground into fine powder, and 100 g of each was measured and kept in amber bottles. Methanol (1 L) was used as the extraction solvent, and the samples were macerated for 72 h. After that, the solution was filtered with Whatman No. 1 filter paper to get a clear extract. The filtrate was evaporated using a rotary evaporator at 40–60 °C and then stored at − 20 °C^10^.

### GC-MS analysis

Chemical composition was studied using a GC-MS system (GCMS-QP2020, Japan) at the Center of Phytochemistry Analysis for Herbal City, Department of Thai traditional Medicine, Faculty of Natural Resources (A405). The analysis used a TR-5MS capillary column (30 m×0.25 mm ×0.25 μm) with split injection (5:1). The injection volume was 1.0 µL, Helium gas (99.99%) was used as the carrier gas at a flow rate of 1.33 mL/min. The injector temperature was set to 200 °C, and the oven temperature was programmed to increase from 100 to 250 °C. The MS conditions were as follows; ionization energy, 70 eV; ion source temperature, 200 °C; and scan range, 40–800 amu. Compound identification was performed by comparing the mass spectra with those in the NIST mass spectral library (National Institute of Standards and Technology) and by comparing chromatographic retention times with previously reported literature data. Relative abundance was expressed as relative area percentage, calculated by peak area normalization as the ratio of each peak area to the total peak area of all detected compounds, multiplied by 100, and with a similarity index (SI) > 80^11^.

### LC-MS analysis

Chemical composition was studied using an ultra-high-performance liquid chromatography–mass spectrometry (UHPLC–MS) system (Thermo Fisher Scientific, Waltham, MA, USA) coupled to a quadrupole mass spectrometer (Surveyor MSQ Plus, Thermo Fisher Scientific, Waltham, MA, USA) equipped with an electrospray ionization (ESI) source operated in positive ion mode. Chromatographic separation was achieved on a reversed-phase C8 column (Phenomenex, Torrance, CA, USA). The mobile phase consisted of (A) 10 mM ammonium formate adjusted to pH 3.5 (Sigma-Aldrich, Darmstadt, Germany) and (B) methanol (Merck, Darmstadt, Germany), delivered at a flow rate of 0.25 mL/min with an injection volume of 2.5 µL^12^. A gradient elution program was applied as follows: 0–2 min, 22% B; 6 min, 40% B; 9 min, 45% B; 12.5 min, 50% B; 14–15.5 min, 80% B; and 16–20 min, 22% B. Mass spectrometric detection was carried out in Selected Ion Monitoring (SIM) mode, monitoring the protonated molecular ion at m/z 399. The probe temperature was set at 450 °C, spray voltage at 4.5 kV, and cone voltage at 100 V^13^.

### Total phenolic content (TPC) analysis

Total phenolic content was measured by the Folin-Ciocalteu method with modification. Gallic acid (Sigma-Aldrich, Darmstadt, Germany) (5–320 µg/mL) was used as the standard. Kratom extracts were prepared at 1 mg/mL. The assay was performed in a 96-well plate by mixing 20 µL of sample extract, 20 µL of Folin-Ciocalteu reagent (Sigma-Aldrich, Darmstadt, Germany), and 100 µL of 7.5% Na_2_CO_3_ (Sigma-Aldrich, Darmstadt, Germany). After 30 min incubation in the dark, the absorbance was recorded at 765 nm using a microplate reader (Tecan, Switzerland). All tests were conducted in triplicate (*n* = 3). The results were calculated from the gallic acid standard curve and reported as mg of gallic acid equivalent per gram extract (mg GAE/g extract)^14^.

### Total flavonoid contents (TFC) analysis

Total flavonoid content was measured by the aluminium chloride method. Quercetin (Sigma-Aldrich, Darmstadt, Germany) (0.78125–50 µg/mL) was used as the standard. Kratom extracts (1 mg/mL) were mixed with 2% AlCl_3_ (Sigma-Aldrich, Darmstadt, Germany) in a 96-well plate (50 µL each). After 15 min in the dark, the absorbance was read at 435 nm (Tecan, Switzerland). Tests were conducted in triplicate (*n* = 3). Results were calculated from the quercetin standard curve and expressed as mg QE/g extract^15^.

### Antioxidant activity

#### 2,2-Diphenyl-1-picrylhydrazyl (DPPH) assay

The antioxidant capacity of the extracts was evaluated using the 2,2-diphenyl-1-picrylhydrazyl (DPPH) radical scavenging assay. A 0.02 mM DPPH solution (Alfa Aesar, Ward Hill, MA, USA) was prepared in 95% ethanol (Sigma-Aldrich, Germany). Serial dilutions of kratom extracts (10–640 µg/mL) were prepared, and 100 µL of each sample was mixed with 100 µL of the DPPH solution in a 96-well microplate. The mixtures were incubated for 30 min in the dark at room temperature, after which the absorbance was measured at 517 nm using a microplate reader (Tecan, Switzerland). Trolox (Sigma-Aldrich, Darmstadt, Germany) (6.25–200 µM) was used as the reference standard. The percentage of radical scavenging activity was calculated, and IC_50_ values were determined from the dose–response curves^16^.

#### 2,2’-azino-bis (3-ethylbenzothiazoline-6-sulfonic acid) (ABTS) assay

The ABTS radical cation was generated by mixing 7 mM ABTS solution (Wako, Osaka, Japan) with 2.47 mM K_2_S_2_O_8_ and incubating the mixture in the dark at room temperature for 12–16 h. Kratom extracts (10–640 µg/mL) 50 µL were then mixed with 100 µL of the ABTS working solution in a 96-well plate. After incubation in the dark for 15 min, absorbance was recorded at 734 nm using a microplate reader. Trolox (6.25–200 µM) served as the reference standard^17^.

#### Ferric reducing antioxidant power (FRAP) assay

The FRAP reagent was prepared by mixing 300 mM acetate buffer (pH 3.6) (Sigma-Aldrich, Darmstadt, Germany), 10 mM FeCl_3_ (Sigma-Aldrich, Darmstadt, Germany), and 10 mM TPTZ (Sigma-Aldrich, Darmstadt, Germany) in a 10:1:1 ratio. Extracts were prepared at 0–1,000 µg/mL. A 20 µL aliquot of each extract was added to a 96-well plate and mixed with 80 µL of FRAP solution. After incubation in the dark for 4 min, absorbance was measured at 593 nm using a microplate reader. Results were calculated from the gallic acid calibration curve and expressed as mg GAE/g extract^18^.

### Anticancer activity

A549 lung cancer cells (CCL-185™) and HeLa cervical cancer cells (CCL-2™) were purchased from the American Type Culture Collection (ATCC, USA) and kindly provided by Prof. Sorujsiri Chareonsudjai, Faculty of Medicine, Khon Kaen University. The KKU213C cholangiocarcinoma cell line was obtained from the Cholangiocarcinoma Research Institute (CARI), Faculty of Medicine, Khon Kaen University, Khon Kaen Thailand. This study was reviewed by the Human Research Ethics Subcommittee of Rajamangala University of Technology Isan, Sakon Nakhon Campus, and was determined to be exempt from full ethical review on December 11, 2024 (Project Code: HEC-04-67-017). The exemption was granted in accordance with the Declaration of Helsinki, the Belmont Report, the CIOMS Guidelines, the International Conference on Harmonization for Good Clinical Practice (ICH-GCP), and the U.S. Code of Federal Regulations.

### Sulforhodamine B (SRB) assay

A549 lung cancer cells (Passage Number (PN) 38), KKU213C cholangiocarcinoma cells (PN 56) and HeLa cervical cancer cells (PN 52) were seeded into 96-well plates at a density of 1 × 10^4^ cells per well and incubated for 24 h. After incubation, the cells were treated with *M. speciosa* extract at various concentrations: (7.8125, 31.25, 62.5, 125, 250, 500, and 1,000 µg/mL^6^. The standard compound Mitragynine (Sigma-Aldrich, Darmstadt, Germany) was tested at concentrations of 3.125, 6.25, 12.5, 25, and 50 µg/mL^19^. Cisplatin (Sigma-Aldrich, Darmstadt, Germany) was tested at concentrations of 0, 0.1, 1, 10, 100, and 1,000 µM in A549 lung cancer cells and HeLa cervical cancer cells. Gemcitabine (Sigma-Aldrich, Darmstadt, Germany) was evaluated in KKU213C cholangiocarcinoma cells at concentrations of 0, 0.1, 1, 10, 100, and 1,000 µM^20^. The cells were kept in a CO_2_ incubator at 37 °C for 24, 48, and 72 h. The cells were then fixed with 10% trichloroacetic acid (TCA) and incubated at 4°C for 40 min, then allowed to equilibrate at room temperature. The cells were stained with 0.4% sulforhodamine B (SRB) in 1% acetic acid and incubated in the dark for 30 min at room temperature. Excess dye was removed by washing with 1% acetic acid/distilled water, and the cells were air-dried. The bound dye was dissolved in Tris base buffer (pH 8). Absorbance was measured at 510 nm using a microplate reader. IC_50_ values were calculated using Calcusyn 2.11 software (Biosoft, Cambridge, UK). All experiments were performed in triplicate (*n* = 3).

### Study of *M. speciosa* (kratom) extract and mitragynine in combination with anticancer drugs

The effect of kratom extract and its main alkaloid, mitragynine, in combination with anticancer drugs was studied in three cancer cell lines: lung cancer (A549), cholangiocarcinoma (KKU213C), and cervical cancer (HeLa). For A549 and HeLa cells, cisplatin was used, and for KKU213C cells, gemcitabine was used. In all models, the anticancer drug concentration was adjusted to IC_25_. Kratom extract was tested at different concentrations depending on the cell line: 0, 62.5, 125, and 250 µg/mL for HeLa cells; 0, 100, 200, and 400 µg/mL for KKU213C cells; and 0, 200, 400, and 800 µg/mL for A549 cells. In addition, mitragynine was tested in the range of 3.125–50 µg/mL combined with the same anticancer drugs. After addition of extract, mitragynine, and drug, the cells were incubated in a CO_2_ incubator (5% CO_2_, 37 °C) for 24 and 48 h. Cell cytotoxicity was evaluated as described above, and the resulting data were used to calculate percentage inhibition. Fold-sensitization = (IC₅₀ of the chemotherapeutic drug alone) / (IC₅₀ of the chemotherapeutic agent combined with kratom extract or mitragynine). This number shows how much kratom extract or mitragynine enhances cancer cells more sensitive to cisplatin or gemcitabine. The Chou–Talalay approach was used to figure out the Combination Index (CI). A CI of less than 1 means that the effects are synergistic, a CI of 1 means that the effects are additive, and a CI of more than 1 means that the effects are antagonistic^10^.

### Western blot analysis of anti-apoptotic protein

Western blotting was performed to evaluate the expression of anti-apoptosis related proteins. A549, KKU213C, and HeLa cells were treated with the kratom extract and anticancer drugs and their combination dose accordingly to 50–60% cell viability for 48 h. For A549 kratom extract 800 µg/mL and cisplatin 62.5 µM, KKU213C kratom extract 400 µg/mL and gemcitabine 7.5 µM, and HeLa kratom extract 62.5 µg/mL and cisplatin 62.5 µM and with combination. After 48 h of treatment, cells were lysed in radioimmunoprecipitation assay (RIPA) buffer containing protease and phosphatase inhibitors. Equal amounts of total protein were separated by SDS-PAGE and transferred onto nitrocellulose membranes. The membranes were cut into smaller strips prior to antibody incubation to conserve antibodies and allow probing of different molecular weight targets. The membranes were blocked in 5% skimmed milk and incubated overnight at 4 °C with primary antibodies targeting Glyceraldehyde-3-phosphate dehydrogenase (housekeeping protein control) and BcL-2 (all at 1:10,000 dilution). After washing, membranes were incubated with horseradish peroxidase (HRP)-conjugated secondary antibodies. Protein bands were visualized using an enhanced chemiluminescence (ECL) detection system. Densitometric quantification was performed using ImageJ software, and protein expression levels were normalized to GAPDH.

### Statistical analysis

The data were expressed as mean ± standard deviation (mean ± SD). IC_50_ values were calculated using Calcusyn 2.11 software (Biosoft, Cambridge, UK). For comparison of two or more groups, variance analysis was done with One-Way ANOVA. The difference between mean values was checked by the Duncan’s multiple range test. Statistical significance was considered at *p < 0.05, **p < 0.01, ***p < 0.001, ****p < 0.001. All analyses were carried out using SPSS Version 27 (IBM, Armonk, NY, USA) and GraphPad Prism Version 10 (GraphPad Software, San Diego, CA, USA).

## Results

### Compound identification by GC-MS analysis

**Fig. 1:**
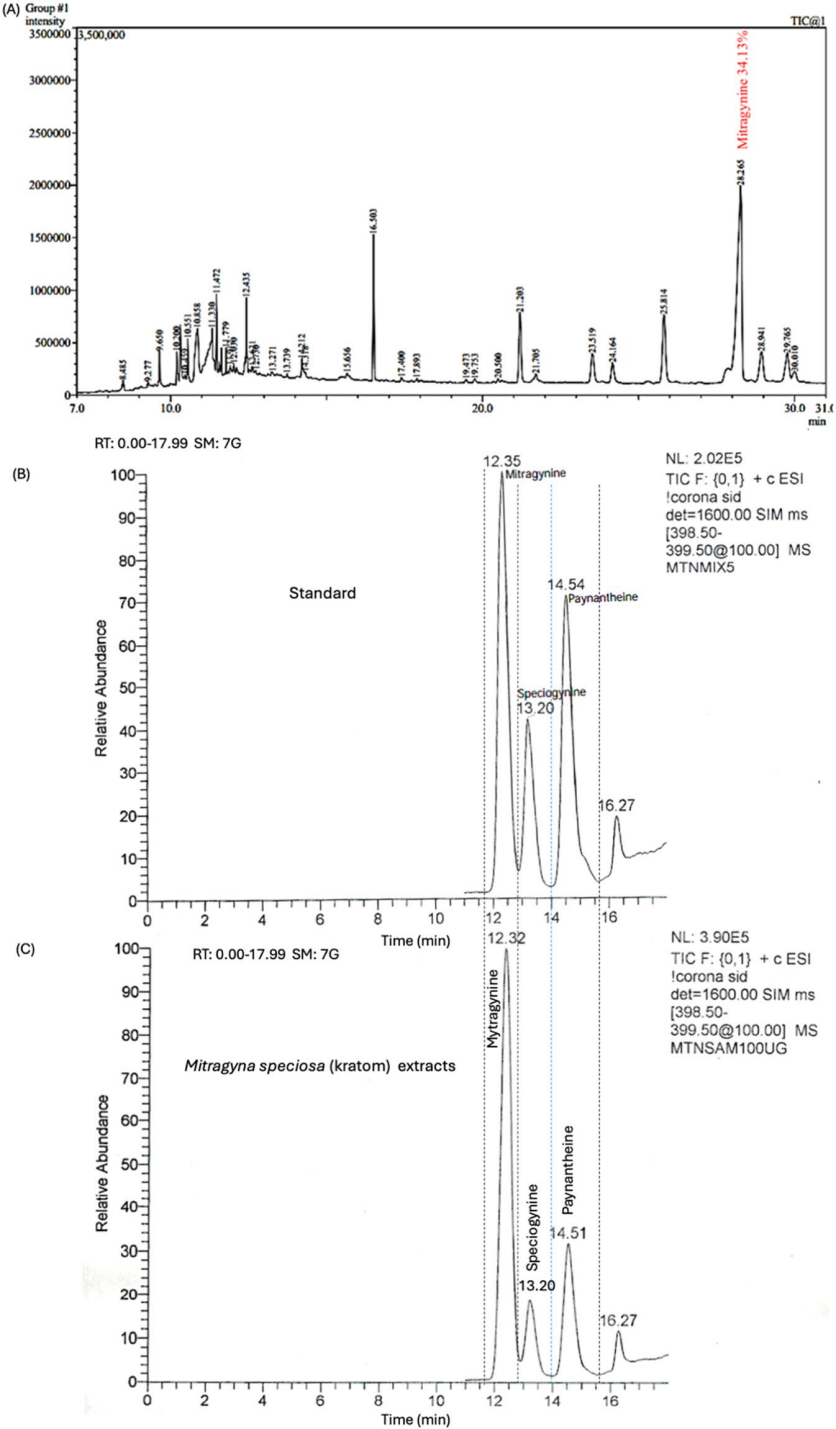
Chromatogram of methanol-extracted *M. speciosa* (kratom) leaves by Gas Chromatography-Mass Spectrometry (GC-MS) (**A**), quantitative analysis of standard using Liquid Chromatography-Mass Spectrometry (LC-MS) (**B**), and analysis of kratom extracts (**C**).

The GC-MS analysis of the methanol extract of *M. speciosa* leaves showed many groups of chemical profile (Fig. 1A and Table 1) such as two alkaloids mitragynine (34.13%), paynantheine (4.07%), campesterol (3.28%), stigmasterol (2.22%), and γ-sitosterol (7.80%), antioxidant compounds including vitamin E (5.66%), β-tocopherol (0.23%), γ-tocopherol (0.33%), and dl-α-tocopherol (1.50%) were also present. The LC–MS analysis of the methanolic extract of *M. speciosa* revealed the presence of major indole alkaloids under positive electrospray ionization conditions. The extracted ion chromatogram monitored at m/z 399 showed a predominant peak corresponding three indole alkaloids were identified: mitragynine 34.13% w/w with Rt 12.32 min as the major compound, followed by paynantheine 4.07% w/w with Rt 14.51 min and speciogynine at 0.86% w/w with Rt 13.60 min (Fig. 1B, C).

### Total phenolic content (TPC) and Total flavonoid content (TFC)

The total phenolic content of *M. speciosa* extracts was measured using gallic acid as the standard. The calibration curve of gallic acid (5–320 µg/mL) exhibited R² = 0.999. The TPC of kratom extract was 265.79 ± 1.11 mg GAE/g extract. The total flavonoid content of *M. speciosa* extract was measured using quercetin as the standard. The calibration curve of quercetin (0.8125–50 µg/mL) also showed R² = 0.999 . The TFC of *M. speciosa* extract was 97.85 ± 0.64 mg QE/g extract (Table 2).

**Table 1:** Detected chemical components in *Mitragyna speciosa* (kratom) extract.

Peak no.	Compounds	Rt (min)	Relative area %
1	Dodecane	8.48	0.55
2	2-Methoxy-4-vinylphenol	9.277	0.43
3	Hexadecane	9.650	1.18
4	2,4-Di-tert-butylphenol	10.2	2.93
5	Phenol, 4-ethenyl-2,6-dimethoxy	10.459	0.60
6	Hexadecane	10.551	1.42
7	1,2,3,5-Cyclohexanetetrol, (1.alpha.,2.beta.,3.alpha.,5.beta.)	10.858	7.43
8	Dodecane, 1-iodo	11.33	3.26
9	Neophytadiene	11.472	2.69
10	Hexadecanoic acid, methyl ester	11.779	1.33
11	n-Hexadecanoic acid	11.779	0.88
12	Hexacosane	12.030	0.85
13	Phytol	12.435	4.21
14	Z-(13,14-Epoxy)tetradec-11-en-1-ol acetate	12.621	0.62
15	Tridecanol, 2-ethyl-2-methyl	12.730	0.34
16	Pentadecanal	13.271	0.32
17	Octadecanal	13.739	0.12
18	Hexadecanoic acid, 2-hydroxy-1-(hydroxymethyl)ethyl ester	14.212	1.23
19	Bis(2-ethylhexyl) phthalate	14.318	0.27
20	Octadecanoic acid, 2,3-dihydroxypropyl ester	15.656	0.35
21	Supraene	16.503	6.07
22	Triacontane	17.400	0.18
23	Oxirane, 2,2-dimethyl-3-(3,7,12,16,20-pentamethyl-3,7,11,15,19-heneicosapentaenyl)-	17.893	0.13
24	beta.-Tocopherol	19.473	0.23
25	gamma.-Tocopherol	19.753	0.33
26	Triacontane, 1-iodo-	20.500	0.20
27	Vitamin E	21.203	5.66
28	Corynan-16-carboxylic acid, 16,17-didehydro-17-methoxy-, methyl ester, (16E,20.beta.)	21.705	0.75
29	Campesterol	23.519	3.28
30	Stigmasterol	24.164	2.22
31	gamma.-Sitosterol	25.814	7.80
32	Mitragynine	28.265	34.13
33	Paynantheine	28.941	4.07
34	Corynan-16-carboxylic acid, 16,17-didehydro-9,17-dimethoxy-, methyl ester, (16E)	29.765	4.06
35	dl-.alpha.-Tocopherol	30.010	1.50
	Total		100

**Table 2:** Total phenolic and total flavonoid contents of kratom extracts.

Samples	Total Phenolic Contents	Total Flavonoid Contents	FRAP	50% inhibition concentration (IC_50_), µg/mL	
				DPPH	ABTS
Kratom	265.79 ± 1.11 mgGAE/g extract	97.85 ± 0.64 mgQE/g extract	414.06 ± 7.30 mg GAE/g extract	211.20 ± 5.67	69.15 ± 0.52
Trolox	–	–		0.34 ± 0.003	0.62 ± 0.002

### Antioxidant activity

The antioxidant activity of *M. speciosa* (kratom) extracts was evaluated by 2,2-Diphenyl-1-picrylhydrazyl (DPPH), 2,2’-azino-bis (3-ethylbenzothiazoline-6-sulfonic acid) (ABTS), and Ferric ion reducing antioxidant power (FRAP) methods. In the DPPH assay, kratom extracts (10–640 µg/mL) showed free radical scavenging with IC_50_ = 211.20 ± 5.67 µg/mL. In comparison, Trolox had IC_50_ = 0.34 ± 0.003 µg/mL, showing significantly higher activity (*p* < 0.05). In the ABTS assay, kratom extracts (10–640 µg/mL) displayed IC_50_ = 69.15 ± 0.52 µg/mL while Trolox showed IC_50_ = 0.62 ± 0.002 µg/mL. In the FRAP assay, kratom extracts (0–1,000 µg/mL) exhibited antioxidant capacity equal to 414.06 ± 7.30 mg GAE/g extract. These results show that kratom extract contains high phenolic and flavonoid content, and demonstrates antioxidant activity. The extract was more active in the ABTS assay than in the DPPH assay, and the FRAP results confirmed its reducing power. This suggests that phenolic and flavonoid compounds in kratom may play a key role in its antioxidant potential.

### Cytotoxicity effects of kratom extract, mitragynine and anticancer drugs

**Fig. 2:**
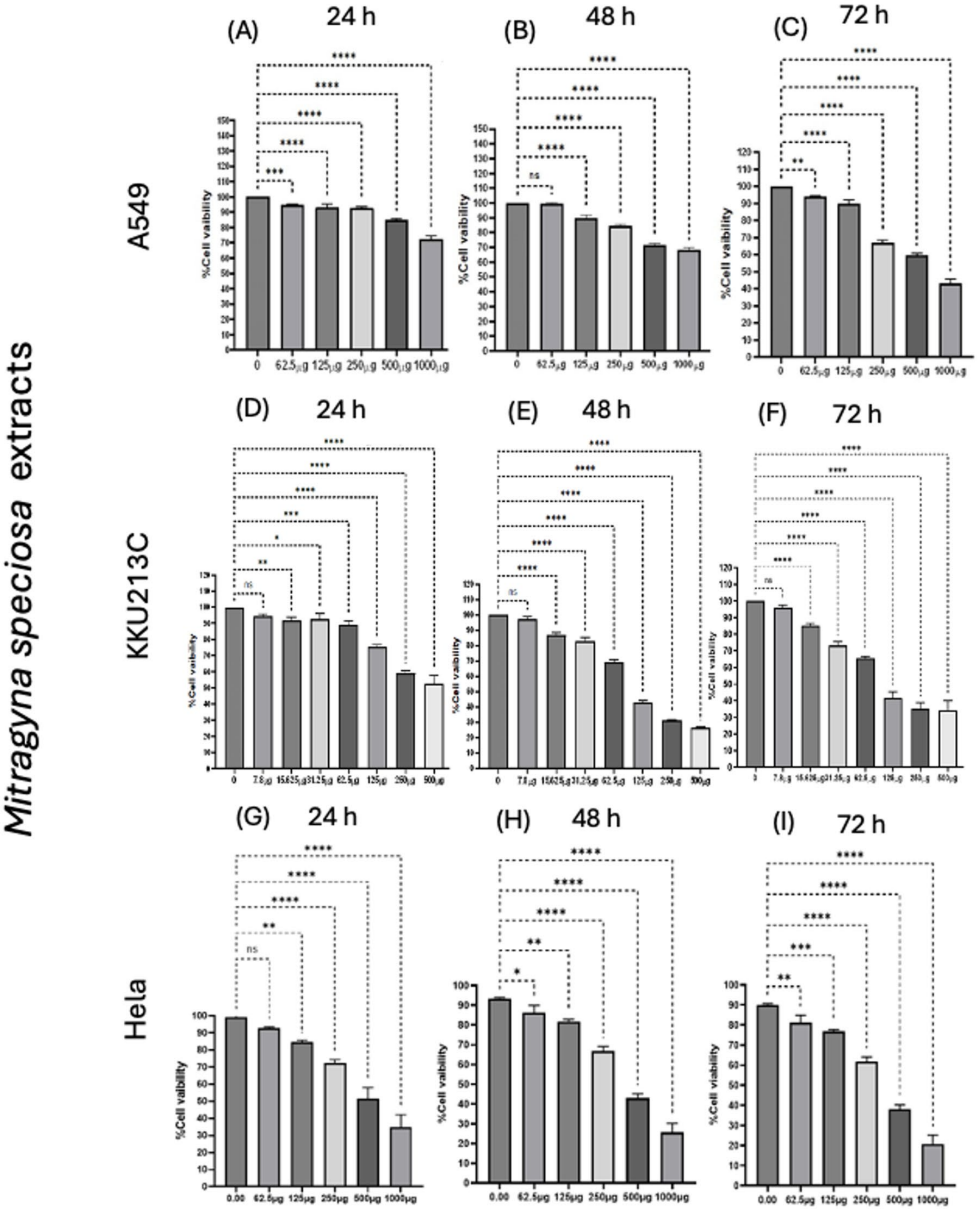
Percentage viability of lung cancer cells (A549) (**A**-**C**), cholangiocarcinoma cells (KKU213C) (**D**–**F**), cervical cancer cells (HeLa) (**G**–**I**), treated with *M. speciosa* (kratom) extract for 24–72 h. * *p* < 0.05, ** *p* < 0.01, *** *p* < 0.001, **** *p* < 0.0001 indicates statistically significant differences between groups.

**Fig. 3:**
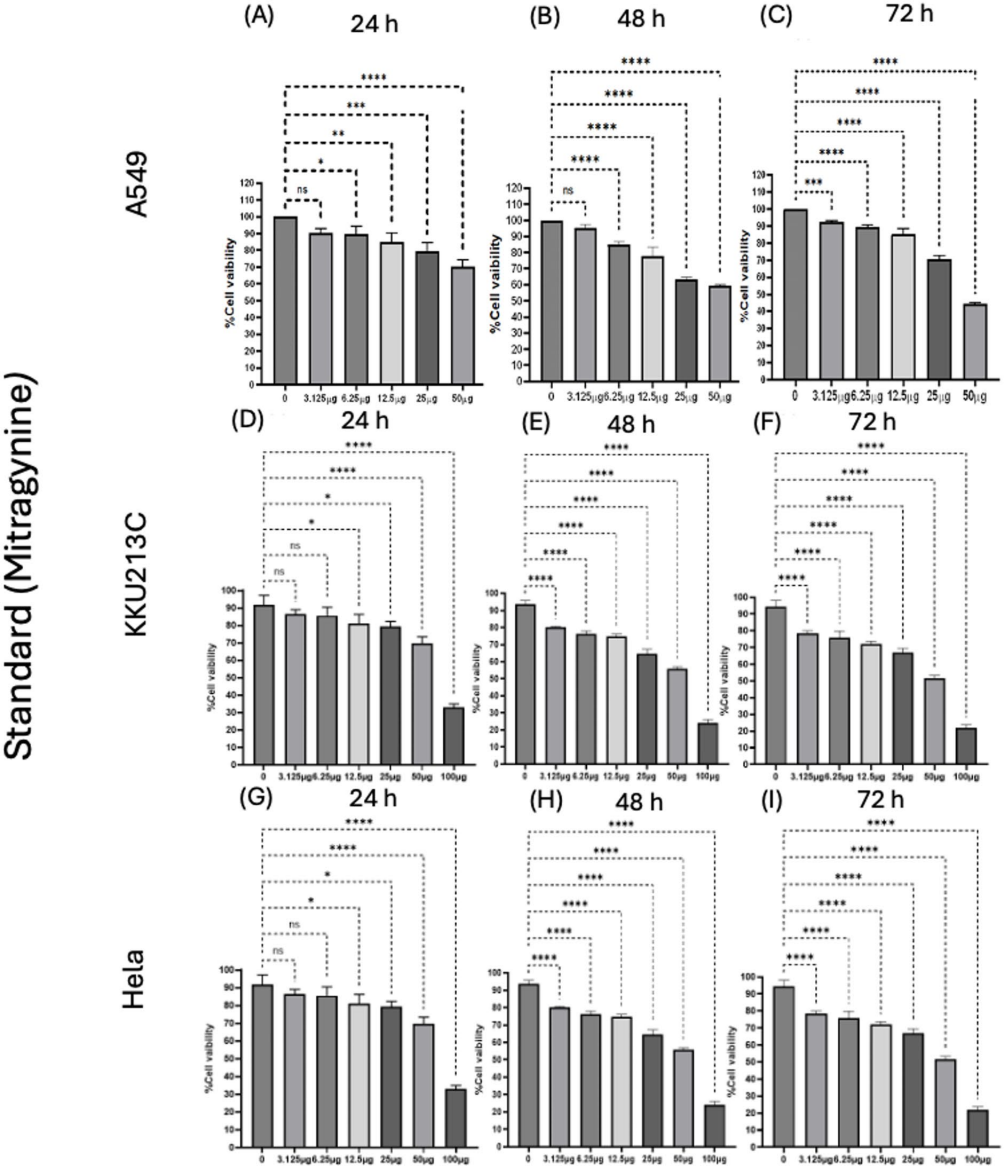
Percentage viability of lung cancer cells (A549) (**A**–**C**), cholangiocarcinoma cells (KKU213C) (**D**–**F**), cervical cancer cells (HeLa) (**G**–**I**), treated with mitragynine for 24–72 h. * *p* < 0.05, ** *p* < 0.01, *** *p* < 0.001, **** *p* < 0.0001) indicates statistically significant differences between groups.

**Fig. 4:**
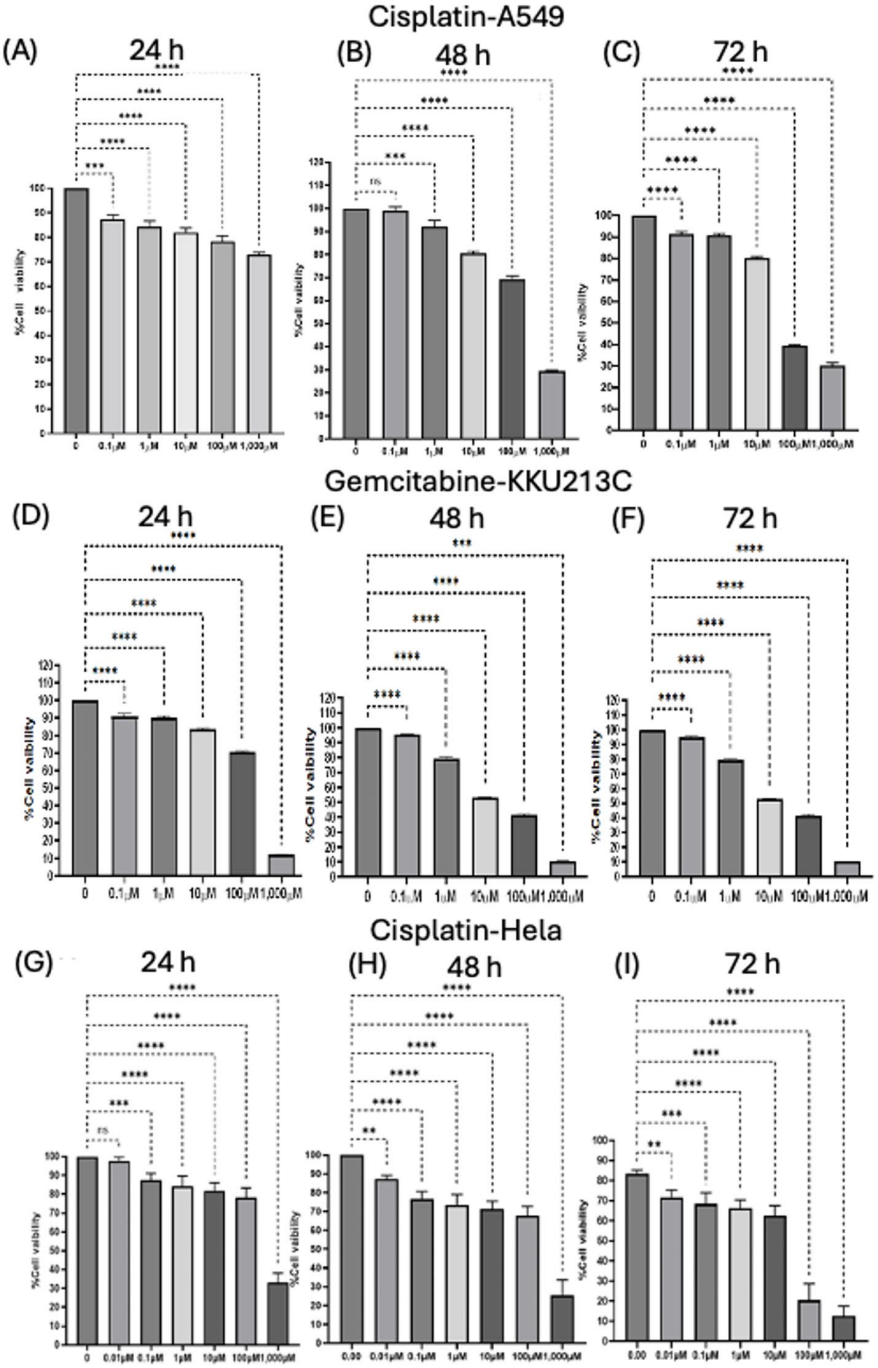
Percentage viability of lung cancer cells (A549)(**A–C**), cholangiocarcinoma cells (KKU213C)(**D**–**F**), cervical cancer cells (HeLa)(**G**–**I**), treated with the anticancer drug cisplatin/gemcitabine over a period of 24 to 72 h. * *p* < 0.05, ** *p* < 0.01, *** *p* < 0.001, **** *p* < 0.0001) indicates statistically significant differences between groups.

Kratom extract inhibited cancer cell growth in a dose- and time-dependent manner (Fig. 2). Among the three cell models, cholangiocarcinoma cells (KKU213C) showed the highest sensitivity to kratom extract, while lung cancer (A549) and cervical cancer (HeLa) cells showed lower susceptibility. The inhibitory effects became stronger with longer incubation time, indicating a clear time-dependent response. Mitragynine also showed significant growth-inhibitory effects on A549, KKU213C, and HeLa cells in a dose- and time-dependent manner (Fig. 3). Notably, mitragynine was more potent than the crude kratom extract, with the strongest antiproliferative activity consistently observed in KKU213C cells at all incubation times. Standard chemotherapeutic agents (cisplatin and gemcitabine) also effectively inhibited cancer cell proliferation in a concentration- and time-dependent manner (Fig. 4). Cisplatin reduced cell viability in A549 and HeLa cells, whereas gemcitabine showed strong inhibitory effects against KKU213C cells. The IC₅₀ values are summarized in Table 3. Overall, both mitragynine and standard anticancer drugs suppressed the proliferation of lung, cholangiocarcinoma, and cervical cancer cells; however, kratom extracts and mitragynine exhibited the strongest inhibitory effect against cholangiocarcinoma cells (KKU213C).

**Table 3:** IC_50_ values of kratom extract, mitragynine, and anticancer drugs in A549, KKU213C and HeLa cancer cells.

Sample	h	IC_50_ (µg/mL)		
		A549	KKU213C	HeLa
Kratom	24	> 1,000^a^	> 500^a^	> 500^a^
	48	> 1,000^a^	117.34 ± 3.89^b^	419.66 ± 20.62^b^
	72	686.77 ± 39.07^b^	109.7 ± 8.76^b^	353.87 ± 87^c^
Mitragynine	24	> 50^a^	26.33 ± 0.15^a^	71.92 ± 4.59^a^
	48	> 50^a^	16.08 ± 0.95^b^	67.06 ± 3.21^a, b^
	72	44.51 ± 1.99^a^	15.14 ± 2.13^c^	58.72 ± 2.37^b^
Cisplatin/	24	> 1,000^a^	125.85 ± 7.12^a^	425.22 ± 56^a^
Gemcitabine	48	248.38 ± 7.83^b^	29 ± 1.39^b^	139 ± 15^b^
	72	108.59 ± 6.45^c^	28.11 ± 1.99^b^	16.50 ± 8.24^c^

^a, b, c^ (*p* < 0.05) indicates statistically significant differences when comparing 24, 48, and 72 h within the same test substance.

### Cytotoxicity effects of kratom extract combined with anticancer drugs

**Fig. 5:**
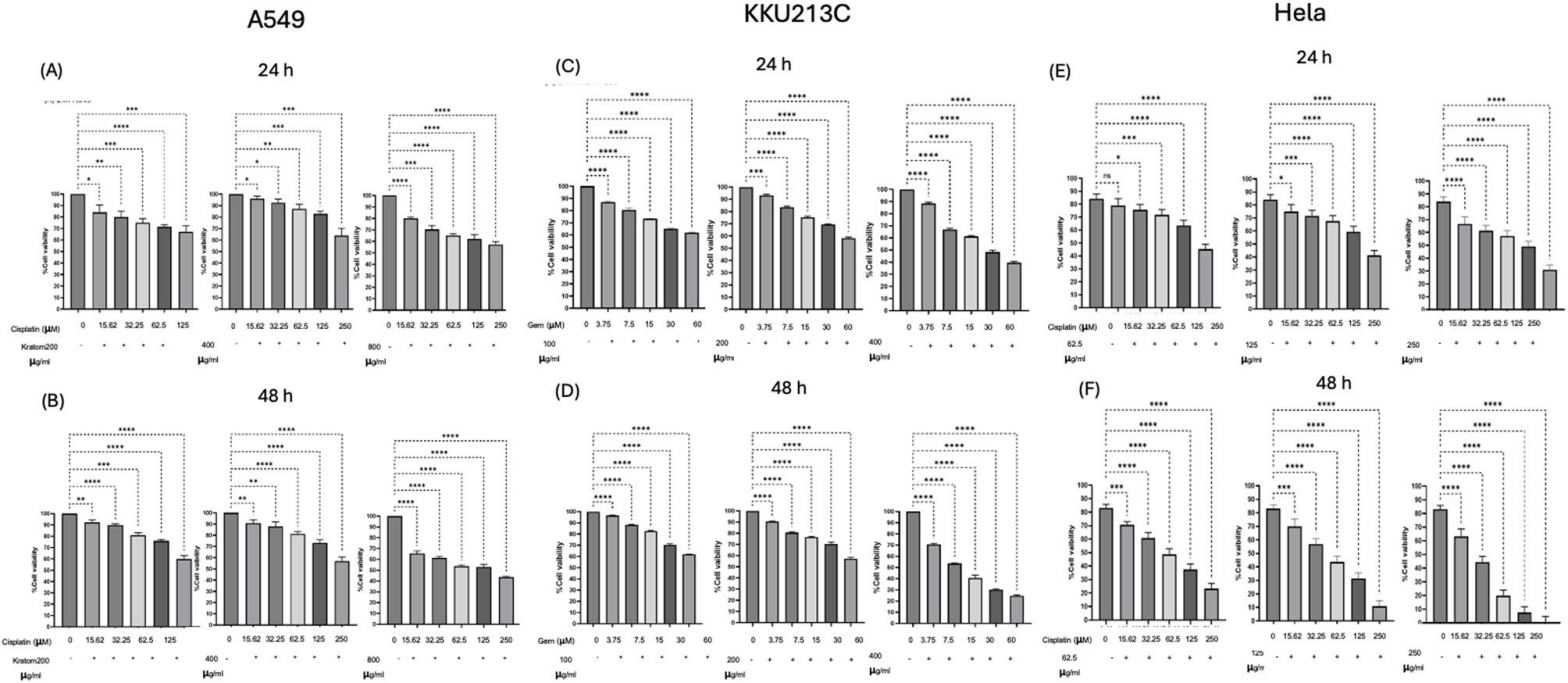
Percentage viability of cancer cells A549 (**A**, **B**), KKU213C (**C**, **D**), and HeLa (**E**, **F**) treated with *M. speciosa* (kratom) extracts in combination with the anticancer drug cisplatin/gemcitabine over 24–48 h. * *p* < 0.05, ** *p* < 0.01, *** *p* < 0.001, **** *p* < 0.0001) indicates statistically significant differences between groups.

The cytotoxic effects of *Mitragyna speciosa* (kratom) in combination with anticancer agents were evaluated across three cancer cell lines (Fig. 5; Table 4). In A549 lung cancer cells, treatment with kratom extract at concentrations of 200 and 400 µg/mL did not yield sufficient cytotoxicity at either 24–48 h to achieve 50% cell inhibition; consequently, fold sensitization and combination index (CI) values could not be established and were denoted as “–” in Table 4. At a higher concentration of 800 µg/mL, there was no effect at 24 h. However, at 48 h, a 3.43-fold sensitization was seen with a CI value of − 0.63, which means that the two substances worked together (CI < 1). In HeLa cervical cancer cells, kratom extract significantly increased sensitivity to cisplatin in a concentration- and time-dependent manner. The combination treatment resulted in significant chemosensitization, evidenced by fold sensitization values between 7.71 and 20.35 and CI values consistently below 1 (− 0.30 to − 1.26), indicating robust synergistic effects. In KKU-M213C cholangiocarcinoma cells, concurrent treatment with kratom extract significantly enhanced chemosensitivity to gemcitabine. At 48 h, kratom extract at 400 µg/mL produced an 8.87-fold sensitization with a confidence interval (CI) value of − 0.01, indicating a synergistic interaction. In general, these results show that kratom extract can make cancer cells more sensitive to standard anticancer drugs. However, the strength of this effect depends on the concentration of the extract, the type of cancer cells, and how long they are exposed to it.

**Table 4:** IC₅₀ values of anticancer drugs (cisplatin or gemcitabine) in A549, HeLa, and KKU213C cancer cells in combination with kratom extract.

Cell line	Concentration µg/mL	IC_50_ Cisplatin-µM (µg/mL)		Fold sensitization		Combination index (CI)	
		24 h	48 h	24 h	48 h	24 h	48 h
A549	200	> 125 (> 37.5)	> 125 (> 37.5)	–	–	–	–
	400	> 125 (> 37.5)	> 125 (> 37.5)	–	–	–	–
	800	> 125 (> 37.5)	55.5 ± 4.5 (16.69 ± 1.37)	–	3.43	–	– 0.63
Hela	62.5	55.12 ± 5.64 (18.09 ± 3.55)	17.04 ± 4.60 (5.02 ± 1.11)	7.71	8.16	– 0.30	– 0.88
	125	48.68 ± 9.19 (14.64 ± 2.77)	13.42 ± 2.27 (3.80 ± 0.73)	8.74	10.36	– 0.39	– 1.00
	250	28.71 ± 5.84 (8.62 ± 1.75)	6.83 ± 1.10 (2.08 ± 0.31)	14.81	20.35	– 0.62	– 1.26
Cell line	Concentration µg/mL	IC_50_ Gemcitabine-µM (µg/mL) 24 h 48 h		Fold sensitization 24 h 48 h		Combination Index (CI) 24 h 48 h	
KKU213 C	100	> 60 (> 15.792)	> 60 (> 15.792)	–	–	–	–
	200	> 60 (> 15.792)	> 60 (> 15.792)	–	–	–	–
	400	> 60 (> 15.792)	26.45 ± 1.32 (6.85 ± 0.25)	–	8.87	–	– 0.01

IC₅₀ values are shown in µM (and µg/mL). “–” means that it cannot be calculated because the treatment did not reach 50% cell death at 24 h. Fold sensitization refers to the extent to which the anticancer drug’s potency increases when combined with kratom extract. Combination Index (CI): CI < 1 indicates synergistic effect; CI = 1 indicates additive effect; CI > 1 indicates antagonistic effect.

### Cytotoxicity effects of mitragynine combined with anticancer drugs

**Fig. 6:**
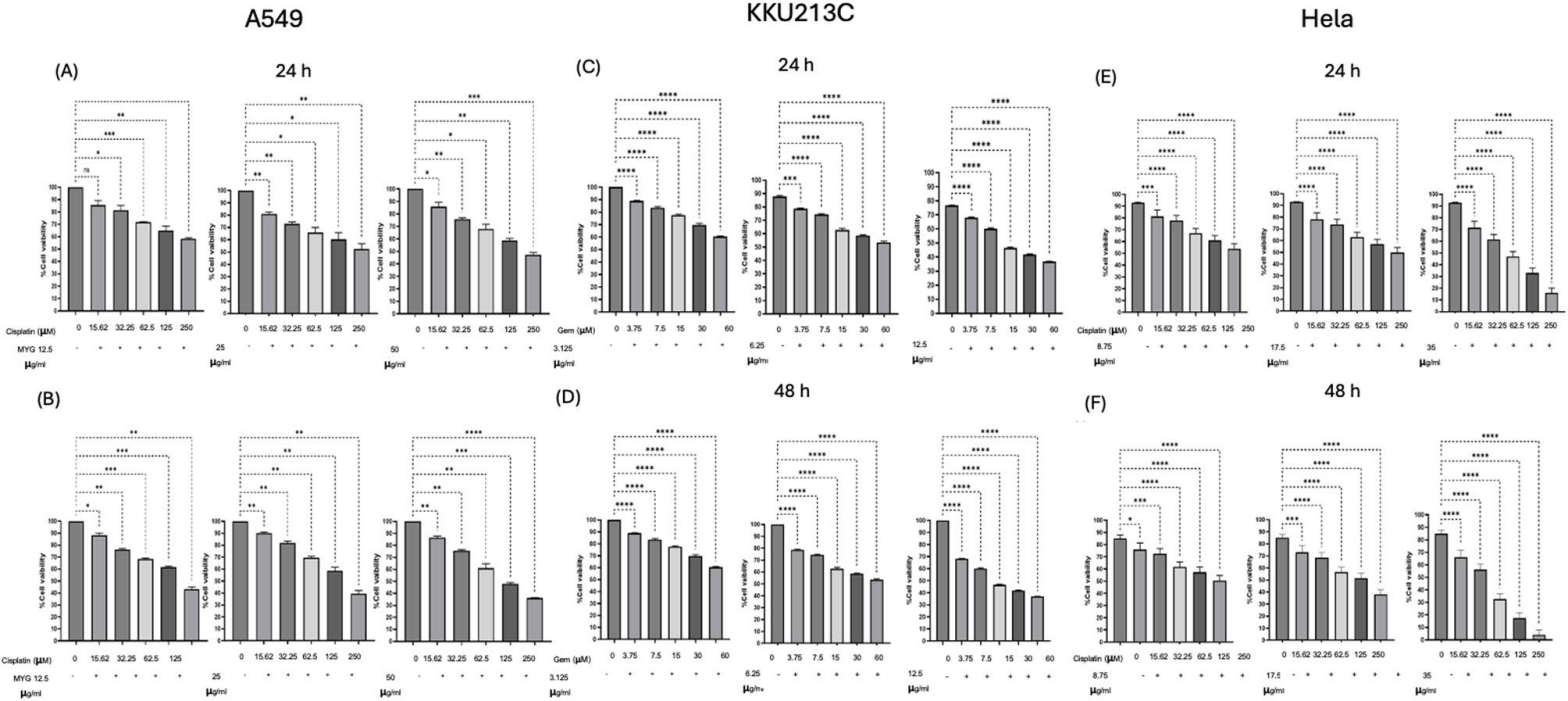
Percentage viability of cancer cells A549 (**A**, **B**), KKU213C (**C**, **D**), and HeLa (**E**, **F**) treated with mitragynine (MYG) in combination with the anticancer drug cisplatin/gemcitabine over 24–48 h. Note: * *p* < 0.05, ** *p* < 0.01, *** *p* < 0.001, **** *p* < 0.0001) indicates statistically significant differences between groups.

The cytotoxic effects of mitragynine in conjunction with anticancer agents were evaluated across three cancer cell lines (Fig. 6; Table 5). In A549 lung cancer cells, treatment with 12.5 and 25 µg/mL of mitragynine exhibited no significant effect at 24 and 48 h, as the treatment failed to induce 50% cell death; consequently, fold sensitization and CI values could not be determined (indicated as “–” in Table 5). Mitragynine at 50 µg/mL caused a small increase in cisplatin activity, with a 2.08-fold sensitization and a combination index (CI) of 0.08, which shows that the two drugs worked together in a mild way. In HeLa cervical cancer cells, the combination with mitragynine exhibited increased efficacy. At 8.75 µg/mL, the combination yielded a 2.48-fold enhancement in sensitization, accompanied by a CI value of -0.12, indicating a synergistic interaction. At higher concentrations (17.5 and 35 µg/mL), the IC values dropped even more, fold sensitization rose to 27 after 24 h, and CI values ranged from below 1 (− 0.33 to − 0.99), showing strong synergistic activity. Mitragynine exhibited notable chemosensitizing effects in KKU213C cholangiocarcinoma cells when administered with gemcitabine at concentrations of 6.25 and 12.5 µg/mL after 48 h. The combination resulted in a 5.7-fold and a 11.45-fold enhancement in sensitivity, respectively. The CI values, which ranged from -0.15 to 0.16, were less than 1, which means that they worked together. In general, mitragynine made cancer cells more responsive to anticancer drugs, but this effect depended on both the dose and the length of time it was given. The synergistic effect was more noticeable in HeLa and KKU213C cells than in A549 cells. Kratom crude extract exhibited moderate effects in A549 cells and more significant effects in HeLa and KKU213C cells (Table 4). Mitragynine, when isolated, showed less effectiveness in A549 cells but greater effectiveness in HeLa and KKU213C cells compared to the impure extract. In summary, both mitragynine and standard anticancer agents impeded the proliferation of lung, cholangiocarcinoma, and cervical cancer cells, with mitragynine exhibiting the most significant effect against cholangiocarcinoma cells.

**Table 5:** IC₅₀ values of anticancer drugs (cisplatin or gemcitabine) in A549, HeLa, and KKU213C cancer cells in combination with mitragynine.

Cell line	Concentration µg/mL	IC_50_ Cisplatin-µM (µg/mL)		Fold sensitization		Combination Index (CI)	
		24 h	48 h	24 h	48 h	24 h	48 h
A549	12.5	> 125 (> 37.5)	> 125 (> 37.5)	–	–	–	–
	25	> 125 (> 37.5)	> 125 (> 37.5)	–	–	–	–
	50	> 125 (> 37.5)	120.43 ± 8.02 (35.73 ± 3.09)	–	2.08	–	0.08
Hela	8.75	> 70 (> 21)	56.08 ± 8.30 (19.41 ± 5.56)	–	2.48	–	– 0.12
	17.5	57.46 ± 9.62 (21.32 ± 10.01)	37.17 ± 11.64 (12.21 ± 3.93)	7.40	3.74	– 0.33	– 0.32
	35	15.75 ± 3.54 (4.46 ± 0.92)	10.19 ± 1.82 (2.83 ± 0.53)	27	13.64	– 0.99	– 0.96
Cell line	Concentration µg/mL	IC_50_ Gemcitabine-µM (µg/mL)		Fold sensitization		Combination Index (CI)	
		24 h	48 h	24 h	48 h	24 h	48 h
KKU213 C	3.125	> 60 (> 15.792)	> 60 (> 15.792)	–	–	–	–
	6.25	> 60 (> 15.792)	30.35 ± 1.68 (8.29 ± 0.35)	–	5.7	–	0.16
	12.5	> 60 (> 15.792)	15.12 ± 0.74 (4.08 ± 0.26)	–	11.45	–	– 0.15

### BCL-2 anti-apoptotic expression

**Fig. 7:**
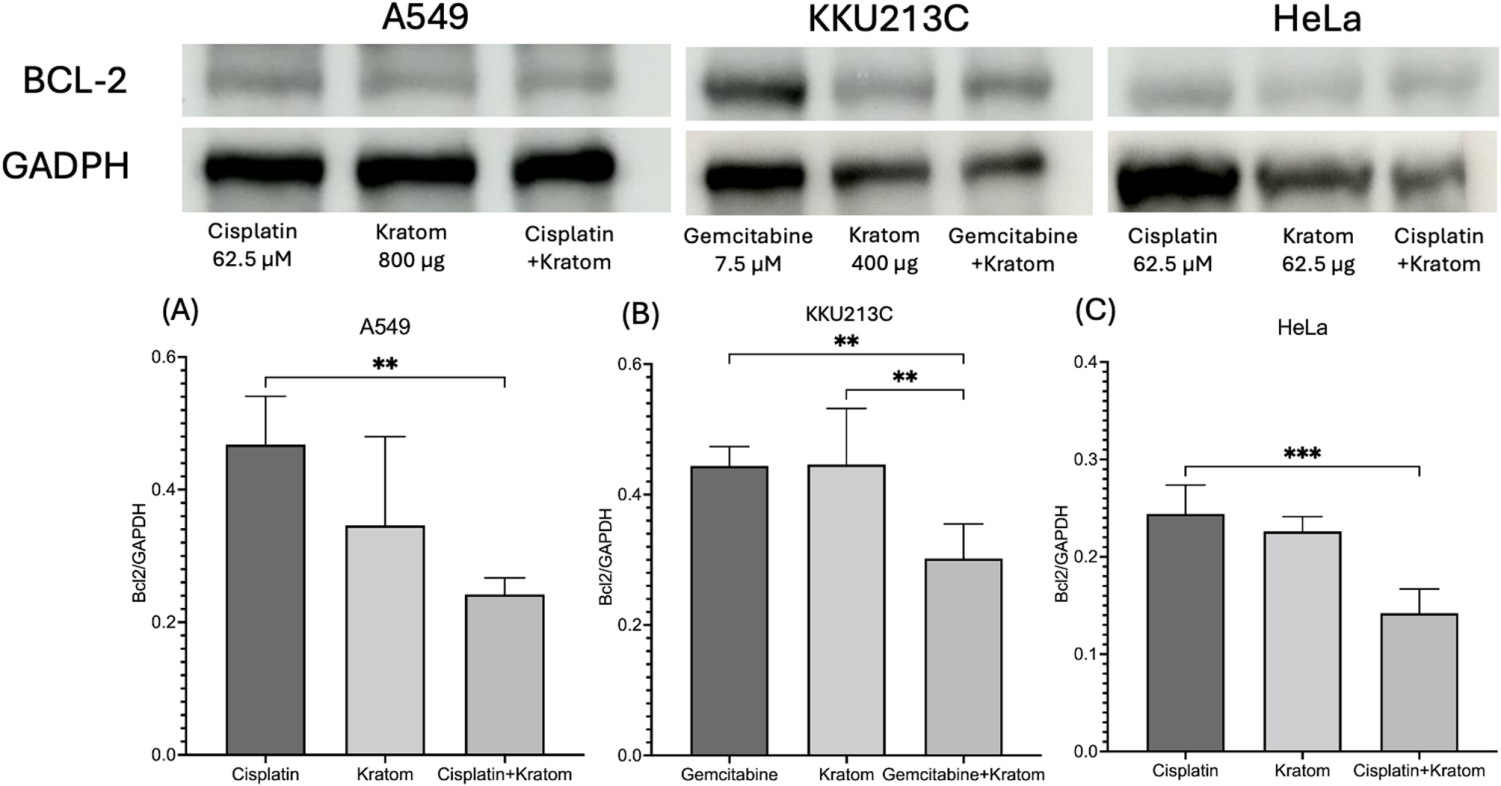
Expression of anti-apoptotic protein BCL-2 by Western blotting and the Bcl-2/GAPDH protein ratio expressed by densitometric analysis of bands. Protein expression levels after treatment with cisplatin, kratom extracts, and gemcitabine alone and their combination were normalized against GADPH and **p < 0.01, ***p < 0.001 significantly different between groups: (**A**) A549 lung cancer, (**B**) KKU213C cholangiocarcinoma, and (**C**) HeLa cervical cancer cell lines.

Western blot analysis was employed to assess the expression levels of Bcl-2, an anti-apoptotic protein, in relation to cellular responses following treatment with the kratom extract and its combination. As depicted in Fig. 7, cells treated with 62.5, 400, and 800 µg/mL of the extract, both alone and in combination with cisplatin (62.5 µM) and gemcitabine (7.5 µM), were analyzed for important regulatory proteins, with GAPDH serving as the internal control. Treatment with kratom extract and its combination led to a notable increase in the expression of the anti-apoptotic protein Bcl-2, which was considerably lower compared to the expression observed with the anti-cancer drug alone across three cell types. Quantitative densitometric analyses of band intensities are displayed in Fig. 7: (A) A549, (B) KKU213C, and (C) HeLa cells.

## Discussion

The study on the extraction of kratom showed the presence of phenolic and flavonoid compounds. However, the values in this work are slightly different from previous study. In their study, the total phenolic content of methanol extract of kratom was 167.43 ± 13.50 mg GAE/g extract, and the total flavonoid content was 347.72 ± 15.97 mg QE/g extract^21^. The antioxidant activity of kratom results showed that both extracts had free radical scavenging activity. These results are in agreement with the report of Parthasarathy et al. (2009) which investigated the antioxidant activity of kratom extracts from aqueous, alkaloid, and methanol fractions^22^. They reported IC_50_ values of 37.08–213.4 µg/mL, showing that the methanol fraction had the strongest antioxidant activity. The IC_50_ values of the ABTS assay were 69.15 ± 0.52 µg/mL; these results are different from the previous study, which tested methanol-extracted kratom and reported an IC_50_ of 4.25 ± 1.59 µg/mL^21^. Additionally, the findings also differ from those previous reports^23^. The FRAP assay results showed that the antioxidant capacity of kratom had IC_50_ values of 414.06 ± 7.30 mg GAE/g extract. These values differ from those reported in a previous study, which investigated the antioxidant activity of a 70% ethanol kratom extract and reported a total flavonoid content of 89.31 ± 1.44 mg quercetin equivalents per gram of extract^23^.

GC-MS analysis of methanol-extracted kratom was done based on retention time. The main compound was mitragynine, which accounted for 34.13% of the total extract confirming mitragynine as the dominant alkaloid in kratom^24^. Corresponding with the findings of previous study, it was indicated that kratom contained elevated levels of mitragynine. GC–MS is a useful way to analyze volatile and semi-volatile metabolites, but it only works for compounds that are volatile and stable at high temperatures^25^. Consequently, non-volatile, high-molecular-weight, or thermolabile compounds may be inadequately represented, partly due to the necessity of derivatization and the impact of chromatographic conditions, including column selection, on compound elution^26^. The GC-MS analysis was corroborated by UHPLC–MS of the kratom extract, which identified three alkaloids: mitragynine as the predominant compound at 34.13% w/w, followed by paynantheine and speciogynine at 0.86% w/w, respectively.

The cytotoxic effects of kratom, mitragynine, and anticancer drugs were tested on lung cancer cells (A549), cholangiocarcinoma cells (KKU213C), and cervical cancer cells (HeLa). After staining with SRB, the control group of viable cells showed strong purple-pink staining. In contrast, cells treated with kratom extract, mitragynine, and anticancer drugs showed lower staining, meaning reduced cell viability. The reduction in cell viability was dependent on both concentration and incubation time. These results are different from the study previous which studied the cytotoxic effects of kratom extracts on Nasopharyngeal cancer cells (NPC/HK1)^10^, breast cancer cells (MCF-7 and MDA-MB-231), and lung cancer cells (A549)^27^. These findings are consistent with previous studies reporting that kratom (*Mitragyna speciosa*) extracts and its major alkaloids exhibit cytotoxic and antiproliferative activity against various cancer cell lines such as colorectal cancer cells (HCT116) and leukemia cancer cells (K562)^9^, providing a scientific basis for further investigation of its potential use in cancer treatment. In the present study, kratom extract and its main alkaloid, mitragynine, induced cell death in lung cancer (A549), cholangiocarcinoma (KKU213C), and cervical cancer (HeLa) cells in a dose- and time-dependent manner. The reduction of cell viability observed in the SRB assay suggests that both the crude extract and mitragynine can interfere with cancer cell growth by triggering apoptotic pathways by decreased BCL-2 expression. Previous studies support these findings, reporting that mitragynine and kratom-derived fractions can activate apoptosis and cell cycle arrest^19^.

These findings explain the anti-proliferative and chemosensitizing effects of *M. speciosa* (kratom) extracts and mitragynine observed in this study. HeLa cervical cancer cells exhibited greater sensitivity than A549 lung cancer and KKU213C cholangiocarcinoma cells, which likely reflects intrinsic biological differences among these cell lines. HeLa cells originate from human papillomavirus (HPV)–associated cervical carcinoma and are characterized by disruption of p53^28^ and retinoblastoma (Rb) signaling pathways^29^, leading to impaired cell-cycle regulation and increased susceptibility to mitochondrial dysfunction and caspase-dependent apoptosis upon exposure to cytotoxic agents. Consistent with this, previous studies have shown that HeLa cells undergo mitochondrial-mediated apoptosis more readily than other cancer cell types when exposed to pro-apoptotic stimuli^30^. The synergistic cytotoxicity likes of saponins and cisplatin, because this anticancer effect was detected in cancer cell lines with both wild-type p53 (A549), inactivated p53 (HeLa) and mutated p53 (SKOV3). Indeed, the independence of p53 would be an advantage of this combination for cancer therapy because p53 is mutated in many types of tumors^31^.

In contrast, A549 cells possess robust DNA damage response and antioxidant defense mechanisms, together with regulation of apoptosis-related pathways, which contribute to their intrinsic resistance to cytotoxic and chemosensitizing agents^32^. Similarly, cholangiocarcinoma cells such as KKU213C are characterized by pronounced chemoresistance mediated by activation of pro-survival signaling pathways, as well as reduced susceptibility to apoptosis. According to the current study, HeLa cells exhibited greater anticancer drug activity enhancement from kratom extract and mitragynine than did A549 and KKU213C cells. When combined, these results demonstrate how mitragynine and kratom alkaloid activity are cell-type-dependent. Previous in silico screening has identified mitragynine and 7-hydroxymitragynine as promising anticancer candidates^33^, while synergistic interactions between kratom alkaloids and conventional chemotherapeutic agents have been reported across multiple cancer models^10^. For nasopharyngeal carcinoma cells (C666-1), mitragynine with cisplatin gave IC_50_ values of 0.8–4.5 µg/mL. These IC₅₀ values were lower than those observed in the present study, but the trend of sensitization is consistent^10^. Mitragynine alkaloids have potential to increase the response of cancer cells to chemotherapy, supporting the idea that mitragynine may act as a chemosensitizer. Together, these results highlight that kratom extract and mitragynine may improve anticancer drug efficacy through synergistic mechanisms. Furthermore, alkaloid extracts from kratom have been reported to enhance the response of A549 lung cancer cells to doxorubicin by 1.3–2.4 times, which is comparable with the findings of this study. At 48 h, the response of A549 cells to cisplatin combined with the standard compound mitragynine increased in a dose-dependent manner, with fold sensitization values of 1.27–2.08, In A549 cells, the crude kratom extract showed moderate synergy at high dose (800 µg/mL), while mitragynine exhibited only limited effects at 50 µg/mL. In HeLa cells, both the extract and mitragynine showed strong synergy with cisplatin. Mitragynine produced higher fold sensitization (up to 27) compared to the extract (maximum 20.35). In KKU213C cells, both kratom extract and mitragynine enhanced the effect of gemcitabine, with mitragynine showing stronger activity (IC_50_ reduced to 15.12 µM, fold sensitization 11.45) compared to the extract (26.45 µM, fold sensitization 8.87). The exact mechanism by which kratom alkaloids enhance the effects of anticancer drugs is not yet fully clear. One hypothesis is that mitragynine potentiates cisplatin through inhibition of mRNA and COX-2 protein expression induced by lipopolysaccharide (LPS), leading to reduced inflammation^34^. Mitragynine may be associated with reduced VEGF signaling, which suppresses tumor angiogenesis, and block the PI3K/Akt signaling pathway to promote apoptosis^35^. In addition, previous studies suggest that mitragynine can induce mitochondrial-mediated apoptosis through activation of caspases and disruption of mitochondrial membrane potential, increase production of reactive oxygen species (ROS), and suppressed anti apoptotic protein BCL-2^36^. These combined mechanisms may explain why mitragynine acts as a chemosensitizer and enhances the cytotoxicity of anticancer drugs in different cancer cell types.

In summary, this study shows that kratom and its main alkaloid, mitragynine, are able to inhibit HeLa cervical carcinoma cells, KKU213C cholangiocarcinoma cells, and A549 lung carcinoma cells. The findings also suggest that kratom may improve the effectiveness of anticancer drugs. More studies are needed to clarify the molecular pathways of apoptosis, to evaluate the effects in animal models, and to begin clinical trials.

## Supplementary Information

Below is the link to the electronic supplementary material.

Supplementary Material 1

## Data Availability

The data supporting the findings of this study are available from the corresponding author upon reasonable request.
