# Supplementary material for: Phytochemical profile and chemosensitizing anticancer activity of Mitragyna speciosa and mitragynine
Source: Sci Rep. 2026 Mar 11;16:13116. doi: 10.1038/s41598-026-43711-5 (PMC13099967; doi:10.1038/s41598-026-43711-5)

Supplementary Information file (Manuscript ID: PRMCM-D-25-01417)

Title: Phytochemical Profile and Chemosensitizing Anticancer Activity of *Mitragyna speciosa* and Mitragynine

GADPH

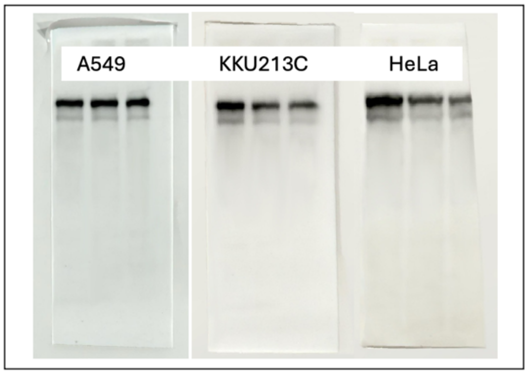

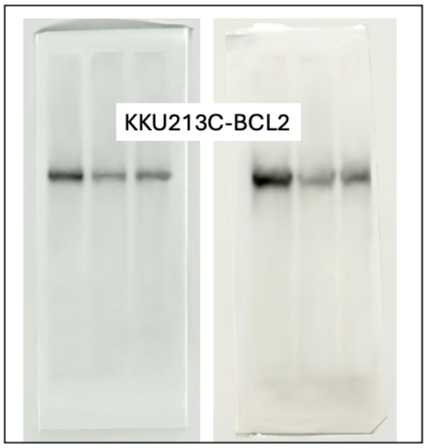

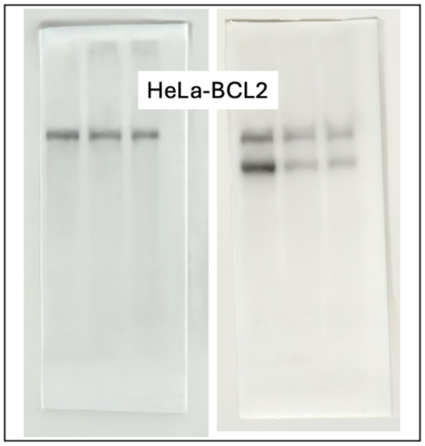

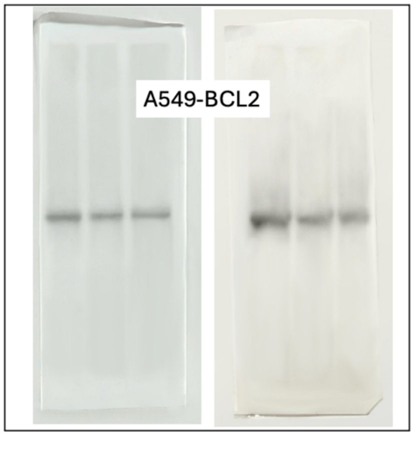

Supplement: Supplementary file 1 — Supplementary Material 1 [file 41598_2026_43711_MOESM1_ESM.pdf]
